# Supplementary material for: Yiai Fuzheng decoction inhibits triple-negative breast cancer by remodeling the immune microenvironment
Source: Front Immunol. 2025 Sep 30;16:1615631. doi: 10.3389/fimmu.2025.1615631 (PMC12518410; doi:10.3389/fimmu.2025.1615631)
Supplement: Supplementary file 2 [file DataSheet1.zip › Supplementary File 2/Treatment/C_vs_D_vs_H/06_Pathway_Analysis/Pathway_Analysis/Sig_Pathways_ID/Butanoate metabolism.pdf]

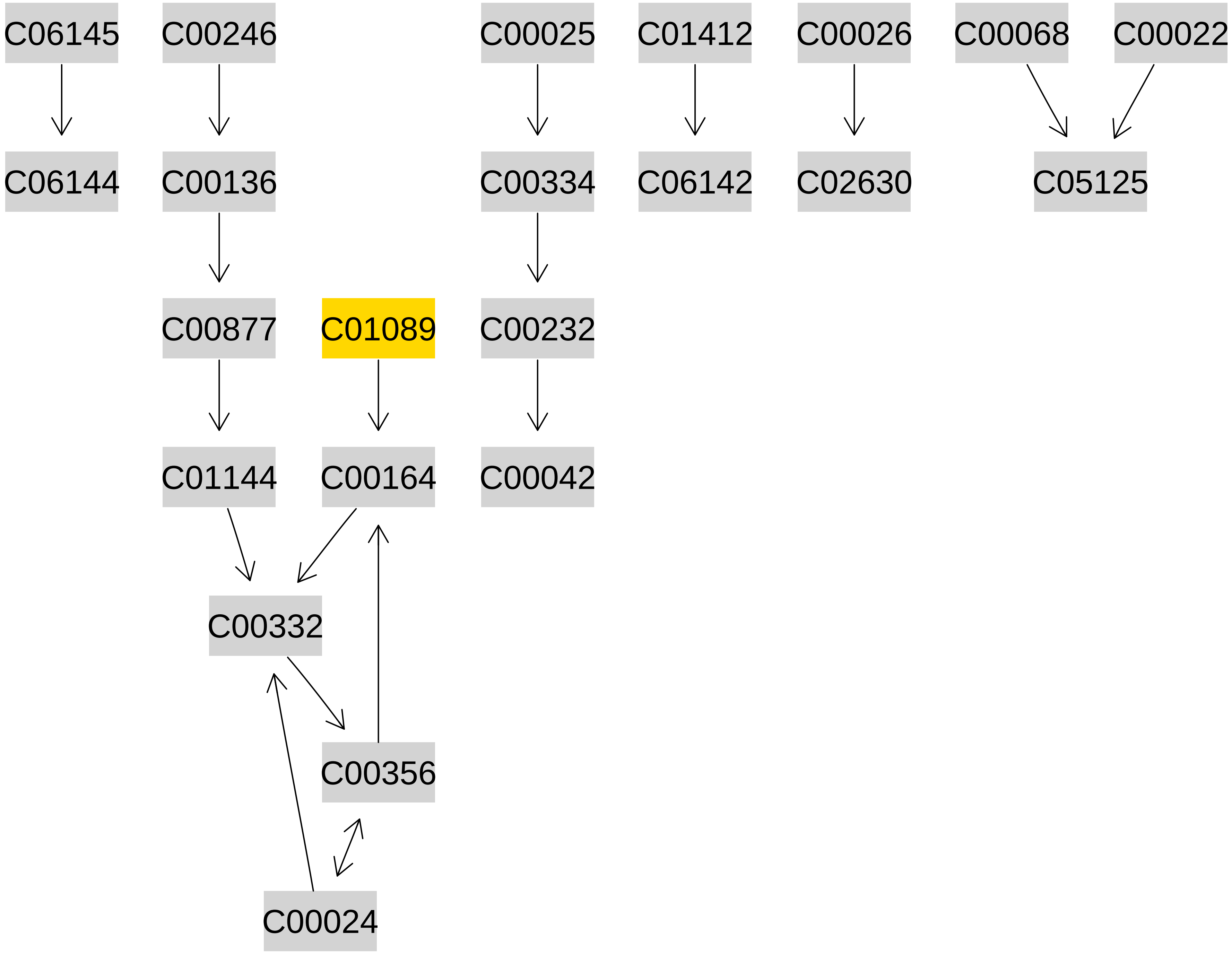
C06145
C00246
C00025
C01412
C00026
C00068
C00022
C06144
C00136
C00334
C06142
C02630
C05125
C00877
C01089
C00232
C01144
C00164
C00042
C00332
C00356
C00024
